# Enhancing medical communication skills through video-recorded peer role-play and a standardized checklist

**DOI:** 10.1371/journal.pone.0343202

**Published:** 2026-02-18

**Authors:** Pallavi L.C, Chinmay Suryavanshi, Krishnamoorthi Prabhu, Archana Chauhan, Kirtana Raghurama Nayak, Ramnarayan Komattil

**Affiliations:** 1 Department of Physiology, Kasturba Medical College, Manipal Academy of Higher Education, Manipal, India; 2 Department of Physiology, Dr. Yashwant Singh Parmar Government Medical College, Nahan, Sirmaur, India; 3 Department of Medical Education, Kasturba Medical College, Manipal Academy of Higher Education, Manipal, India; King Abdulaziz University Faculty of Medicine, SAUDI ARABIA

## Abstract

**Objective:**

Developing communication skills early during medical training requires structured opportunities for practice, observation, and reflection. This study integrated video-recorded peer role-play with a standardized checklist to foster communication skill competencies among first-year medical students.

**Methods:**

A communication skills module was designed for 250 first-year MBBS students as part of the Attitude EThics COMmunication (AETCOM) course. The intervention integrated video-based submission of peer role-play of doctor‒patient interviews guided by a standardized, culturally contextualized checklist (Observation-Based Communication Skills Checklist; OCSC). The entire module was managed through the institutional learning management system (LMS). Student perceptions were gathered via a validated questionnaire and two focus group discussions (FGDs).

**Results:**

Of 241 valid submissions, 70% met expectations, and 28% exceeded them. Student feedback was overwhelmingly positive, with 87% agreeing that peer role-play was an effective learning method. A majority (91%) reported that the OCSC-guided video assignment provided a structured and reflective learning experience. Thematic analysis of FGDs revealed that checklist-guided role-play was helpful for skill acquisition, video assignment consolidated learning, and faculty feedback was crucial for improvement.

**Conclusion:**

The integration of video-recorded peer role-play, a standardized checklist, and an LMS provides a scalable and effective framework for teaching, assessing, and reinforcing foundational communication skills in early-phase medical learners.

## Introduction

Effective communication is fundamental to building trust, ensuring patient safety, and improving health outcomes. Training in communication skills during medical education prepares future doctors to interact empathetically, convey information clearly, and navigate complex clinical scenarios. Effective communication skills, as one of the core competencies required of health care professionals, were introduced as a component of the Attitude EThics COMmunication (AETCOM) course in the competency-based undergraduate medical curriculum in India by its regulatory body, the National Medical Commission (NMC), in 2019 [1].

### Structured learning approach for communication skills training

Acquisition of effective communication skills requires early and sustained training with active student involvement. A structured learner-centered approach promotes the development of these essential competencies by promoting repeated practice, reflection, and active participation [2]. In this context, blending faculty led instruction with opportunities for learners to engage with learning resources, practice communication skills, and reflect on their performance shifts the focus from teacher-driven instruction to learner-centered approaches. This approach enhances learner engagement and fosters lifelong learning habits essential for effective doctor–patient interactions.

### Role-play for the development of communication skills

Experiential learning methods, especially role-play, are more effective than traditional didactic approaches such as lectures for learning communication skills [3]. In peer role-play, students have the opportunity to take on the role of a doctor while also gaining valuable experience by stepping into the role of a patient. Role-play offers an opportunity to practice and rehearse communication skills similar to employing a simulated patient, without the significant time and cost associated with training patients [4]. The acquisition of communication skills during peer role-play can be facilitated by providing descriptive feedback on performance by faculty, reviewing one’s own practice via video/audio recording, and observing others in practice [5].

### Technology to support learning in medical education

Integrating technology into medical education has become increasingly essential, especially for promoting active and flexible learning [6]. Recent advancements in digital technology and mobile devices have rendered video production both feasible and economical for students [7]. Video-based assessments have long been employed in medical education, with educators assigning video production tasks to facilitate learning [8]. Peer role-play through student-generated video assignments provides a self-directed, adaptable learning experience that extends beyond traditional classroom boundaries. The integration of video-recorded peer role-play empowers students to engage in reflective practice by reviewing their recorded interactions [9]. This structured learning model fosters a deeper understanding and mastery of communication skills, as students can analyze their performance, receive feedback from peers and faculty, and make targeted improvements. These student-generated videos have been proven to bolster both learning and assessment [10].

### Using standardized checklists for communication skills training

Traditional peer role-play presents several challenges. It requires students to independently manage their preparation, execution, and reflection, which can be time-consuming within tight academic schedule. Without a structured framework, students may struggle to maintain a focus on the primary learning objectives, potentially diluting the educational goals as they shift their attention toward performance aspects. The inherent variability in individual role-play performance can result in inconsistent outcomes, making it challenging to achieve uniform learning experiences [4].

To address these issues in traditional peer role-play, a structured and standardized method is needed to ensure consistency in both assessment and learning experiences for all learners [11]. By providing a clear framework, students can concentrate on key aspects of communication skills in their role. This approach not only helps maintain consistency but also supports reflective learning, enabling students to identify their strengths and areas for improvement based on the feedback received [12]. The incorporation of a checklist to evaluate learner proficiency in skill acquisition enhances the objectivity and reliability of the skill assessment. When used as part of the learning process, a checklist also functions as a structured guide, directing learners to the key steps required to perform the technique correctly and achieve effective skill acquisition. The Kalamazoo checklist is a standardized tool for assessing communication skills; however, it is not suitable for first-year students because its criteria do not align with their educational context [13,14]. This study adopted and explored the use of the observation-based communication skills checklist (OCSC), which is an educational and culturally contextualized communication skills assessment tool specifically designed for early learners in the Bachelor of Medicine and Bachelor of Surgery (MBBS) program [15]. It provides a clear and objective framework for both students and faculty, ensuring consistent and unbiased feedback. This structured approach not only streamlines the evaluation process but also facilitates effective and equitable assessment across diverse student groups.

- While technology-supported approaches such as video-based peer role-play show promise for enhancing communication skills training in medical education, there is limited evidence on their structured implementation and assessment in early-phase medical learners. In particular, the use of standardized, observation-based checklists to guide learning and evaluate communication competencies in this context remains underexplored. This study was therefore undertaken to examine the educational impact of a structured, video-recorded peer role-play intervention guided by the OCSC among first-year medical students. Accordingly, the study addressed the following research questions

What is the effect of a structured, video-recorded peer role-play intervention on first-year medical students’ doctor–patient communication skills, as assessed using the OCSC?

How do first-year medical students perceive the usefulness and effectiveness of video-recorded peer role-play, guided by the OCSC, for learning and assessing doctor–patient communication skills?

## Materials and methods

### Study population, setting and educational intervention

The study involved 250 first-year MBBS students from the Batch of 2023–24. Data were collected between 22^nd^ April 2024 and 31^st^ August 2024. The AETCOM course is a longitudinal component of the medical curriculum, comprising 27 learning modules that are taught from the first to the final professional year. In Phase I, students engage with five of these modules, which are integrated throughout the MBBS program with other modules to ensure comprehensive development. Module 1.4 in the AETCOM course introduces the foundations of communication in the first professional year. A Communication Skills Module was designed with a focus on teaching, learning, and assessing communication skills.

Faculty from the Department of Physiology, trained in an AETCOM workshop by experts from the National Medical Commission and with extensive experience in teaching AETCOM, taught and assessed the Communication Skills Module. A sensitization session was conducted by the principal investigator to familiarize the faculty with the use of the OCSC and the guidelines for marking the checklist. This 2-hour session, conducted as a workshop, included role-plays with simulated patients to provide hands-on experience in applying the checklist effectively.

The Communication Skills Module was conducted with the following activities:

a) Large Group teaching in a classroom session
b) Video-recorded peer role-play conducted as an asynchronous learning activity using the LMS
c) Assessment and feedback

### Large group teaching in a classroom session

MBBS Phase I students from batches 2023–24 were introduced to the basic principles of communication through an introductory lecture and interactive discussions in a classroom setting. This was followed by a three-hour session on role-playing, facilitated by five instructors, with students divided into small groups of 40–50. These sessions aimed to develop students’ interviewing skills, enabling them to communicate with patients in a respectful, nonthreatening, nonjudgmental, and empathetic manner.

Validated scripts depicting both good and bad communication behaviors were prepared to guide role-play activities in small groups. These two versions of role-play were enacted by the students volunteering as doctors and patients. The students were tasked with identifying clues related to the verbal, nonverbal, and paraverbal components of communication behavior in role-playing using OCSC. A debriefing session was conducted at the end of the session, during which doubts and queries were addressed.

### Video-recorded peer role-play in self-directed learning session

After the classroom sessions, the students were asked to volunteer and form pairs for a peer role-play. Each pair was tasked with creating and submitting an individual assignment on the LMS: a three-minute video recording of a peer role-play demonstrating communication skills. A dedicated ‘Assignment’ activity was created for video submissions, allowing students to upload their files directly. Each faculty member was assigned 25 pairs to facilitate the preparation of video of the peer role-play through the LMS. Faculty facilitators monitored group activities and student participation daily, offering necessary instructions and feedback.

For the role of doctor, the students were guided with a specific focus on interviewing a patient presenting to the community health facility (S1 File) and were instructed to use the OCSC checklist to direct the interview. A list of validated case scenarios (S2 File) was provided for students to choose from and prepare for the role of the patient. Each student, acting as a doctor, conducted the interview after selecting a case from a set of predetermined scenarios, with volunteer peers playing the role of patients for the role-play that was video recorded. They were guided to prepare scripts that would structure the interview, although they were discouraged from acting through verbatim scripts. The students were informed that the video peer role-plays would be graded using a rubric based on the OCSC. The students had two weeks to submit their assignments. The students used their smartphones to record the video of the interview. LMS was used to share learning resources, create discussion boards and assignments, facilitate student submissions, and enable faculty evaluation.

### Assessment and feedback

Five faculty members assessed the video assignments on peer role-plays. focusing on how well students adhered to the key communication points. The faculty used the integrated ‘Grading’ feature in the LMS, which was configured with the OCSC-based rubric, to provide both numerical scores and written descriptive feedback. Communication skills in the videos were graded via a standardized rubric based on the OCSC and additional scoring for the quality of video and audio. The total score for the video-based assignment was 35 points. Learners received scores and decisions, categorized as ‘exceeds expectations’, ‘meets expectations’, or ‘below expectations’, along with descriptive feedback for improvement. It was performed within three weeks after the submission of assignments.

After the assignments were assessed on the LMS, the graded rubric was published, and students received individualized descriptive feedback on the video-recorded peer role-plays. Students were encouraged to reflect on the faculty feedback and their own observations from watching their video on the basis of the assessment. For the assigned 25 pairs, the faculty provided feedback during a plenary session, highlighting both areas of strength and those requiring improvement.

### Perception of the students on the Communication Skills Module

Feedback from learners was collected through a newly developed communication skills questionnaire, which contained 15 closed-ended questions and two open-ended questions. A five-point Likert scale was used for rating the responses to the closed-ended questions. The reliability of the questionnaire was tested with 18 students, who were then excluded from the final survey. Two in-person focus group discussions (FGDs) were conducted, each comprising six participants. After obtaining written informed consent and permission for audio recording, the discussions began. The participants were selected on the basis of their willingness to participate.

A focus group discussion guide was developed to gather in-depth feedback from first-year medical students on their experiences with the Communication Skills Module. The guide was designed to elicit detailed responses regarding the effectiveness of the integrated video-based peer role-play in learning interviewing skills. The discussion aimed to explore students’ perceptions of the role-play’s impact on their learning, the clarity and utility of the OCSC, and the overall effectiveness of the communication skills module. Additionally, the guide aimed to identify any challenges faced by students, gather their suggestions for improvement, and elicit their reflections on how the module influenced their development of communication skills. Written informed consent was obtained for the survey and FGD participation, and any identifiable information was anonymized prior to data analysis. The survey was administered using Microsoft Office forms, and participants responded anonymously. To ensure a safe and non-judgmental environment, FGDs were conducted after completion of grading and assessment, and participation was voluntary. Discussions focused on the learning process rather than individual performance, and all data were sufficiently redacted during analysis to prevent identification of participants.

**Fig 1:**
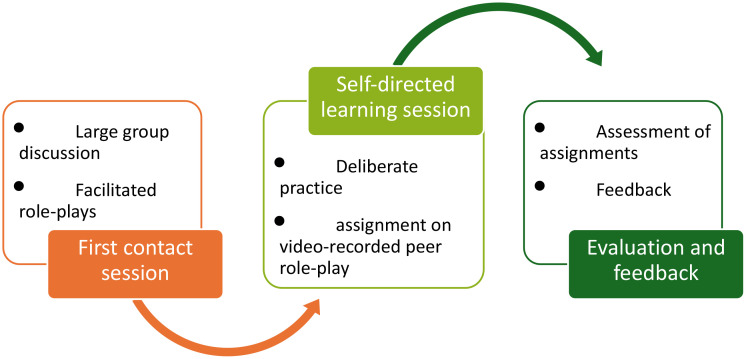
Study design.

The study design is presented in Fig 1.

### Study tools

The OCSC, a standardized checklist validated in the Indian medical education context, is tailored for early-phase learners unfamiliar with clinical situations such as diagnosis and follow-up. It encompasses four sections of medical interviewing and eight domains. The four sections of medical interviewing are as follows: setting up an interview, opening an interview, conducting an interview, and closing an interview. The eight domains in the checklist include rapport building, introduction, clarity, attention, responsiveness, empathy, support, and summarization. It encompasses verbal, nonverbal, and paraverbal aspects of communication behavior and employs a dichotomous scale for scoring, with ratings of 0 or 1 across various parameters.

### Statistical analysis

The SPSS (version 18.0) statistical package and Jamovi software were utilized for data analysis, with thematic analysis conducted on FGD transcripts to identify and finalize themes [16]. Quotes were carefully selected to align with these themes.

## Results

**Fig 2:**
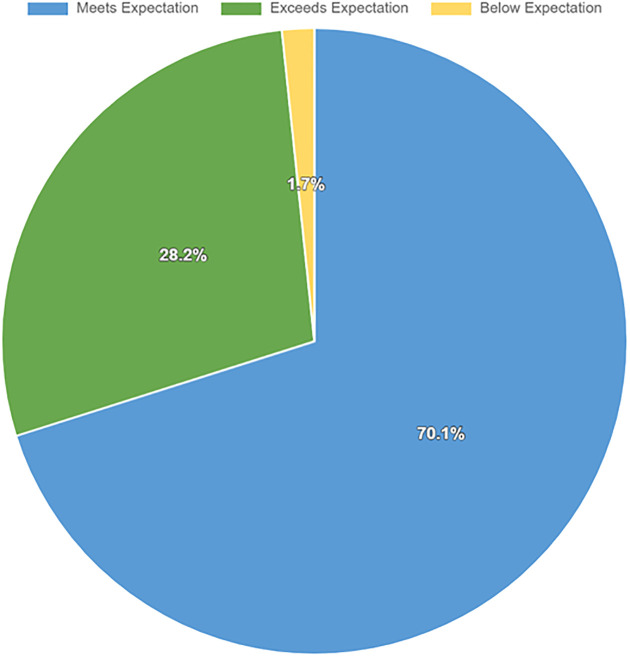
Performance outcomes of the video-recorded peer role-play assignment.

Of the 250 students enrolled, 244 successfully submitted their video-based peer role-play assignments. Of these submissions, 241 were deemed valid for evaluation, whereas three were excluded because of incomplete audio or video data. The performance outcomes, as depicted in Fig 2, are categorized as follows: exceed expectations (28%), meet expectations (70%), and below expectations (2%).

The reliability of the Communication Skills Questionnaire tool was high, with a coefficient of 0.96. Among the students, 223 responded to the questionnaire via Microsoft Office Forms. The responses of the students to the communications skills module are summarized in Table 1. Eighty-seven percent of the students acknowledged that peer role-play was effective for practicing and learning the communication skills required as a doctor. Additionally, 91% agreed that the OCSC checklist provided valuable insights for preparing video-recorded peer role-play. A substantial 92% agreed that the lessons learned would enhance their future patient interactions during clinical years. The LMS was deemed user-friendly by 79% of the participants, facilitating straightforward video assignment submissions.

**Table 1:** The feedback of students for the Communication Skills Module.

Item	Agree (Score 5 & 4) N (%)	Neutral (Score 3) N (%)	Disagree (Score 1 & 2) N (%)
**The sessions helped me understand the importance of communication in doctor patient relationship**	209 (94%)	10 (5%)	4 (2%)
**The peer roleplay used in the session allowed me to learn communication skills**	194 (87%)	22 (10%)	7 (3%)
**The explanation provided by the facilitator on the principles of communication was sufficient and clear**	205 (92%)	14 (6%)	4 (2%)
**The OCSC checklist offered valuable insights into the steps necessary for making medical interview video**	202 (91%)	19 (9%)	2 (1%)
**The OCSC checklist helped me pay attention to cues related to nonverbal communication during the task of making the video on interview**	203 (91%)	16 (7%)	4 (2%)
**There was adequate time for practice and submit assignment**	197 (88%)	13 (6%)	13 (6%)
**The LMS was easy to explore and submit the assignment video**	176 (79%)	25 (11%)	22 (10%)
**All the activities in this Communication skills module were adequately spaced for facilitating my learning**	196 (88%)	23 (10%)	4 (2%)
**The duration of the entire Communication skills module on foundations of communication was adequate**	202 (91%)	16 (7%)	5 (2%)
**I enjoyed attending the Communication skills module**	168 (75%)	44 (20%)	11 (5%)
**I actively participated in the interactive discussions**	169 (76%)	44 (20%)	10 (4%)
**The session’s learning would facilitate my interaction with patients during my clinical postings next year.**	205 (92%)	15 (7%)	3 (1%)
**I liked the idea of video assignment using peer role-play for testing my communication skills**	163 (73%)	38 (17%)	22 (10%)
**I wish to have such innovative assignments in the future AETCOM sessions.**	156 (70%)	36 (16%)	31 (14%)
**I will recommend this learning experience to my peers**	176 (79%)	32 (14%)	15 (7%)

*OCSC, Observation-based communication skills checklist; LMS, Learning management system; AETCOM, Attitude Ethics and Communication*

Table 2 summarizes the responses of 241 students to the OCSC checklist items, which assess the impact of a video-based peer role-play assignment on communication skills. In the ‘Interview Setup’ section, the vast majority donned white coats (99.59%) and ensured a comfortable environment for the patient (96.27%), thereby fostering a nonjudgmental and nonthreatening atmosphere (86.72%). However, only a minority demonstrated respect for the patient’s culture and beliefs (34.85%) and for privacy and confidentiality (54.36%). Most students addressed patients by name (77.59%) and inquired about the reason for their visit (96.68%). During the interviews, nearly all participants (99.59%) used layman’s terms and aligned their vocal tone with the spoken content (88.38%); however, only 44.4% adequately explained medical terminology.

**Table 2:** The responses of students to the rubric based on the OCSC checklist for a video-recorded peer role-play assignment in terms of communication skills.

Observed behavior	Number of students scored with positive response n (%) (N = 241)	Number of students scored with negative response n (%) (N = 241)
**SETS-UP AN INTERVIEW**		
1. Neat appearance and wears white coat.	240 (99.59%)	1 (0.41%)
2. Ensures comfort of patients with respect to the environment.	232 (96.27%)	9 (3.73%)
3. Shows respect for the patient’s privacy & confidentiality.	131 (54.36%)	110 (45.64%)
4. Shows respect for the patient ‘s culture, beliefs.	84 (34.85%)	157 (65.15%)
5. Establish trust by nonthreatening & nonjudgmental behavior	209 (86.72%)	32 (13.28%)
**OPENS AN INTERVIEW**		
1. Addresses the patient by name.	187 (77.59%)	54 (22.41%)
2. Ask for a reason to visit.	233 (96.68%)	8 (3.32%)
3. Provide an overview of the interview’s purpose.	84 (34.85%)	157 (65.15%)
**CONDUCTS AN INTERVIEW: C.A.R.E.S.** **Clarity – Communicates clearly and precisely**		
1. Uses everyday language.	240 (99.59%)	1 (0.41%)
2. Uses appropriate vocabulary.	237 (98.34%)	4 (1.66%)
3. Explains medical terms (if used).	107 (44.4%)	134 (55.6%)
4. Matches voice tone and intonation with the verbal content.	213 (88.38%)	28 (11.62%)
**Attention – Shows interest and listens with patience**		
1. Active listening with minimal interruption, avoids self-touching and nonpurposive movements.	230 (95.44%)	11 (4.56%)
2. Frequent vertical head nods.	207 (85.89%)	34 (14.11%)
3. Occasional Vocalization (uhm. Hmm… umm).	134 (55.6%)	107 (44.4%)
4. Appropriate length of eye contact is comfortable to the patient.	212 (87.97%)	29 (12.03%)
5. Forward lean facing the patient with uncrossed arms	194 (80.5%)	47 (19.5%)
6. Matches facial expressions with verbal content.	192 (79.67%)	49 (20.33%)
**Responsiveness – Encourages the patient to express himself/herself.**		
1. Allow the patient to narrate illness.	240 (99.59%)	1 (0.41%)
2. Asks open ended questions (avoids double barrel & negative questions).	238 (98.76%)	3 (1.24%)
3. Allow wait time for patients to respond.	239 (99.17%)	2 (0.83%)
4. Probe understanding and reinstate important points.	142 (58.92%)	99 (41.08%)
5. Checks accuracy of the statement by getting the patient’s approval.	78 (32.37%)	163 (67.63%)
**Empathy – Shows compassion with values**		
1. Responds to the patient’s emotions.	203 (84.23%)	38 (15.77%)

*OCSC, Observation-based communication skills checklist; C.A.R.E.S., Clarity, Attention, Responsiveness, Empathy, Support*

In terms of attention and active listening, these skills were exhibited by 95.44% of the students, with appropriate eye contact maintained by 87.97%. The students allowed patients to describe their illness (99.59%) and provided them with time to respond (99.17%). Probing for understanding and verifying the accuracy of statements was less common, at 58.92% and 32.37%, respectively. Emotional responsiveness and verbal reassurance were noted in 84.23% and 97.1% of the interactions, respectively. However, recognition of patient coping strategies and understanding barriers to compliance were evident in 45.64% and 29.46% of the submissions, respectively. In concluding the interviews, while most students offered guidance on the next steps (98.34%), only 55.19% inquired about unresolved issues.

### Qualitative analysis

#### Thematic analysis with study participants yielded three distinct themes.

### ......Theme 1: Peer role-play guided by the OCSC checklist was helpful for learning communication skills.

Peer role-play provides experiential learning for learners to practice their interviewing skills. The checklist enabled first-year students to focus on the essential steps necessary for conducting a doctor‒patient interview. It taught them the different layers of interaction, including nonverbal cues, from starting the conversation to closing the interview.

*“With our interview (peer role-play), we learned as a doctor, how we should present ourselves in front of the patient, wearing a white coat, should be listeners first, and I think it’s important to listen to the patient to communicate, should be mindful.”* (FGD-1)

*“The checklist was very good. It had a lot of points that it was very thorough, starting from.... how a doctor is supposed to address a patient when he enters the room to the point where he’s even leaving”.* (FGD-1)

*“Checklist gives us definitive guidelines to okay so when a patient comes, you’re not supposed to do this, the entire point of the checklist it reinforces all of these.*.” (FGD-1)

*“However, one thing about body language I just want to stress again is that if it does not come naturally to someone, this checklist will be life saver for them because they can learn from this* document.” (FGD-2)

### Theme 2: Video assignment of peer role-play consolidates the learning of communication skills.

This was the first attempt by students to conduct and record an interview as a doctor. They noted that the conversation dynamics between friends and between a doctor and patient were vastly different. Scriptwriting, role-playing, and video preparation facilitated a deeper understanding of the interview process, making it a memorable and instructive experience.

*“There’s a lot of differences between a conversation between friends and a conversation between a patient and a doctor. In addition, I think that made the line very clear. Like, especially because we were doing a video, but it gave it a very professional setting, like, okay, meet these checkpoints, make sure these criteria are being fulfilled.”* (FGD-1)

*“I remember the first short of video that we made, we were like, Oh, we are not maintaining eye contact, you are cutting me off here or you are not saying this line here. In addition, then the second time, it was much better; we were conscious of which all criteria we did not fulfill.” (FGD-1)*

*“AETCOM assignment that we had on making the video was the most fun and engaging thing.”. (FGD-2)*

*“It made me realize if you ask two questions at the same time, they will not be able to answer both the questions properly; they will focus on one question at a time, yeah they do stick if you make a video about, it will stick.” (FGD-2)*

### Theme 3: Faculty and peer feedback aided in the learning of communication skills.

Peer role-play supported collaborative learning. The feedback from faculty and peers facilitated preparation for role-play and submission of assignments. The rubric provided after grading enabled the students to identify areas for improvement in future interviews.

*“We learned from each other. When we practiced with each other (role-play), our partner told us what went OK and not.”* (FGD-1)

*“Each time we observe our video, we know what we can do better”* (FGD-1)

*“We got the graded rubric after evaluation of the assignment, how many steps we adhered to and what we have missed.”* (FGD-2)

## Discussion

As India’s medical education system continues to evolve under the National Medical Commission’s competency-based framework, developing effective and scalable training models for core skills such as communication is a national priority. This study introduced first-year medical students to competencies in communication skills through peer role-play, which were assessed through individual video-based assignments. In the peer role-play videos, the students acted as doctors, whereas peers functioned as patients, allowing the students to practice their interviewing skills. We utilized the OCSC checklist, a standardized and validated instrument suitable for early learners, to guide peer role-play and also, to evaluate communication skills. Soft skills such as communication require time to develop; therefore, their early integration into the medical curriculum is recommended [17].

While a previous study reported the use of role-play as part of a communication training program for first-year medical students, it was primarily used as an educational method for students to learn medical interviewing skills without structured assignments for reinforcement [11]. To our knowledge, this is the first study to introduce video-recorded peer role-play with a standardized, observation-based communication skill checklist to enhance structured learning and assessment of competencies in communication skills among first-year medical students.

### Peer role-play supports the acquisition of communication skills

Eighty-seven percent of the students agreed that the peer role-play allowed them to experience interviewing skills, and the video assignment based on peer role-play further allowed them to rehearse and reflect on their newly acquired skills, prompting introspection and self-assessment prior to uploading their final videos (Theme 2). This positions the assignment not merely as an assessment *of* learning, but also as a powerful form of assessment, where the repetitive process of scripting, recording, and self-review becomes the primary learning activity. This finding is consistent with the literature, which shows that peer role-play is an effective method for learning communication skills [4,5]. Additionally, the practice of recording interview videos and submitting them as assignments have led to learning gains in teamwork, communication, technical skills, problem-solving, and leadership skills [18]. According to Kolb’s experiential learning and Kneebone theory-based approach, structured role-play enables learners to engage in focused practice in safe, controlled, learner-centered environments [19,20].

### Technology for an effective learning experience

Technology can enhance effective learning, and digital, web-based technologies are transforming delivery, access, and experience of learning. When students create their own multimedia, such as videos or podcasts, it provides a novel way for learners to disseminate information. Beyond the primary goal of communication training, this process concurrently develops crucial digital literacies essential for modern physicians, including video production, online submission protocols, and the ability to interpret digital feedback from faculty via the LMS. This aligns with research showing that such assignments foster not only content-specific skills but also teamwork, time management, organization, and planning skills [18,21]. Video-based assignments allow for richer analysis of communication skills through observation and differentiation of effective versus less effective communication interactions.

### Standardized checklists as a framework for teaching and assessment

In our study, each video was evaluated using the OCSC checklist, which also guided the students in designing and recording their interviews for peer role-play. The checklist breaks down the interview process into discrete, measurable steps, providing a scaffold for skill development and a tool for faculty to assess skill acquisition [22]. The participants reported that the OCSC checklist helped them focus on the essential steps required for conducting a medical interview (Theme 1), with 91% of the students finding it valuable for their peer role-play video preparation. A prior study implemented an online video peer-based assessment system for nursing students to bolster their communication skills, utilizing the YouTube platform for logging views, ratings, and peer feedback [23]. In a study by Dohms et al., first-year medical residents in a primary care setting presented a prerecorded interview in a real-life setting to a group of peers, subsequently receiving feedback from colleagues and two facilitators [24].

### Role of faculty in skills training

In learner-centered instructional designs, faculty serve as facilitators guiding students through the process of learning experiences that are challenging and authentic, designing innovative assignments, and harnessing the strengths of collaborative learning [25]. In our study, the faculty designed a unique assignment that encouraged learners to practice communication skills, with videos of interviews submitted as evidence of learning. Through these activities, students are encouraged to self-assess and make appropriate corrections. Participating in peer role-play and reviewing their own video fostered self-reflection and feedback from peers (Theme 3). Sharing the rubric with personalized descriptive feedback after evaluation highlights learning gaps, allowing students to improve their subsequent training performance [26].

### Strengths and gaps in communication skills

When the videos were evaluated with the checklist, the students generally scored positively across all domains (Table 2). They excelled in areas such as ensuring patient comfort, actively listening to patients, allowing them to narrate their illness, and offering supportive assurances. Nevertheless, there is room for improvement in aspects such as respecting patients’ cultural beliefs, verifying statement accuracy with patient confirmation, and empathizing with patients’ compliance barriers. Even experienced healthcare professionals frequently encounter challenges in identifying barriers to treatment adherence and one solution that has been suggested is to utilize motivational clinical interviewing techniques [27]. Our early-phase learners likely need more practice and in-depth interviews to navigate complex patient conversations.

While this study was situated in the context of first year, the pedagogical model is highly transferable to other professional years in medical education for further development and reinforcement of communication skills. The core principles of using student-generated video, guided by a standardized rubric and facilitated through an LMS, provide a flexible and scalable framework for authentic assessment across various fields. This broad applicability emphasizes the study’s contribution not only to medical training but also to the wider field of technology-enhanced higher education.

### Limitations and strengths

Although peers provided feedback on the skills performed, this study did not incorporate a rubric for peer assessment, which could be a limitation. The number of hours allotted for this module, the large volume of students, and time constraints associated with the duration of teaching blocks hindered the introduction of formal peer assessment. Additionally, the duration of the video assignment may constrain learners from fully exploring and addressing all facets of the interview. A potential challenge was the initial hesitancy that some early learners may experience when role-playing on camera. While the study adopted a learner-centered approach to communication skills training, self-directed learning as a distinct educational method was not evaluated.

The strength of our study lies in the alignment of learning objectives, instructional activities, and assessments at the ‘how’ level, enabling learners to acquire fundamental competency in communication skills. New medical students were guided through experiential learning, which encompassed the design, writing, and recording of clinical interviews through peer role-play.

## Conclusion

This study successfully demonstrates that an integrated approach combining video-recorded peer role-play, a standardized checklist (OCSC), and an LMS is an effective and well-received method for developing foundational communication skills in first-year medical students. This approach fosters a learner-centered design, with a framework for authentic assessment, to develop competencies such as communication skills essential for medical graduates. This scalable, technology-supported pedagogical design represents a valuable contribution to the AETCOM curriculum, offering a strong template for similar skill-based training across medical institutions in India.

## Supporting information

S1 File Task for the role as doctor. (DOCX)

S2 File Task for the role as patient. (DOCX)
