# Supplementary material for: Enhancing medical communication skills through video-recorded peer role-play and a standardized checklist
Source: PLoS One. 2026 Feb 18;21(2):e0343202. doi: 10.1371/journal.pone.0343202 (PMC12915916; doi:10.1371/journal.pone.0343202)
Supplement: S1 File — (DOCX) [file pone.0343202.s001.docx]

Task for the student as doctor:

- You are a medical student posted at the Community health centre
- The medical officer is held up with administrative work and has asked you to see the next patient
- You are expected to collect history from the patient focusing on why the patient has visited the health centre, what are the presenting complaints including questions about background information such as family and occupational history
- During conversation, it can be helpful to identify the patients belief, concerns and worries about the illness
