# Supplementary material for: Enhancing medical communication skills through video-recorded peer role-play and a standardized checklist
Source: PLoS One. 2026 Feb 18;21(2):e0343202. doi: 10.1371/journal.pone.0343202 (PMC12915916; doi:10.1371/journal.pone.0343202)
Supplement: S2 File — (DOCX) [file pone.0343202.s002.docx]

**Case 1: Patient Role**

- You are a 28-year-old graphic designer with one-month history of experiencing upper abdomen discomfort.
- Your job, at a busy design firm has been hectic and you are regularly working 10hr days even going to the extent of skipping meals or eating on-the-go.
- Recently, you have also experienced some upper stomach burning particularly after meals, along with bloating and occasional nausea. The pain is worse when you are stressed up, and you have occasional nighttime acid reflux.
- You have arrived at the community health centre today because the symptoms have become more frequent and are starting to interfere with your work. You’re concerned that it might be something serious like an ulcer.
- The receptionist tells you there will be an appointment in about 20 minutes with the physician. For now, you will be interviewed by a first-year medical student for prior assessment.
- You are worried that your symptoms might be caused by something more serious, but also think maybe its just stress and eating at odd hours. Express your concerns at a "moderate" level with verbal/written signs and silent cues (e.g. sighing, rubbing belly/ribcage, talking slower or with quiet pauses). If the medical student validates your concerns —"It seems like you’re scared of what’s causing these symptoms. So let us ensure we get deep into it 🡪respond with thoughtful cheerfulness.
- You have a fear of the tests to be done, especially an endoscopy after Googling that these can be painful and uncomfortable. But you won't say that until the medical student directly inquiries about your fears or anxieties over upcoming procedures.
- You have tried over the counter antacid medications, which helped to reduce symptoms but temporally. You have never seen a doctor about the problem before & you do not have any major past medical history.
- You can either build the rest of your backstory or use your own experiences.

**Case 2: Patient Role**

- You are 35-year-old and work as an office manager; you have noticed yourself getting more tired over the last six months.
- You’ve noticed that you’ve been gaining weight despite no significant changes in your diet or exercise routine. You also suspect that it has become unusually cold in general, even when others around you are comfortable.
- Lately, you've experienced some hair thinning and dry skin as well which has added to your concern. You have also observed that your memory and concentration aren’t as sharp as they used to be.
- You visit the community health centre today, because at this point the symptoms are starting to affect your work and daily life. Based on internet search, you suspect that there may be a problem with your thyroid.
- The receptionist has said that, you would be seen by a consultant in approximately 15 minutes. And also, a first-year medical student would be interviewing you before the examination.
- You suspect (or have a diagnosis of) hypothyroidism, but you are concerned about what that means in terms of treatment. Show a moderate level of concern enough to voice and show worries (like speaking slowly/tired looking/rubbing on hands/worry over own symptoms). If the medical student validates your concerns --> “It sounds like you’re anxious about what these symptoms mean for you and why they are occurring. Talk about it, let’s discuss this further to understand what’s going on." – respond with cautious relief
- You know that thyroid issues can be hereditary, but you are not certain if any of the individuals in your family had a problem with their thyroids. You are also scared that you may have to take pills for the rest of your life. Unless the medical student has inquired about what's troubling you or known if you know anything regarding thyroid problems -you will not bring this up.
- You know that thyroid issues can be hereditary, but you are not certain if any of the individuals in your family had a problem with their thyroids. You are also scared that you may have to take pills for the rest of your life. Unless the medical student has inquired about what's troubling you or what you know about thyroid conditions -you will not bring this up.
- You can either make up the rest of your background or use your own experiences

**Case 3: Patient Role**

- You are a 47-year-old accountant who was diagnosed with Type-II Diabetes recently.
- Two weeks ago, you were diagnosed after experiencing increased thirst, frequent urination, and fatigue for several months. When you first noticed it, you initially thought these symptoms were due to stress and to work too long, then testing your blood sugar levels with routine clinical lab reveals a high level.
- You are feeling overwhelmed by the diagnosis and have no idea how to manage your condition. You have been advised some information about Diabetes and metformin tablets has been started for you, but you are still trying to come to terms with the lifestyle changes you need to make.
- You are in the community health centre today for follow up visit, to discuss your treatment plan and address any questions. You are informed that the consultant would there in 10 mins but first, you need to complete a subjective assessment from the first-year medical student before meeting the consultant.
- You worry about how diabetes will change your life, specifically your diet and the need to monitor your blood sugar regularly. Express a moderate level of concern through verbal and non-verbal cues (e.g., speaking hesitantly, looking worried, sighing occasionally). If the medical student acknowledges your concerns – "“It sounds to me like you are trying to deal with this diagnosis, and it is highly stressful. Let’s go through what you’re worried about, and we can figure out a way to handle this both." –reply with a mix of relief and uncertainty.
- You are getting really worried about making diet changes as you love sweet foods and do not know how much or little to restrict. You are also concerned about the potential complications like losing your vision or even having to take insulin for years, perhaps life. But you are not going to mention this unless the medical student asks you about your concerns or what do know in regards with diabetes
- You have no family history of diabetes and you've always been fine, but for the past 10 years or so it's your weight that has gone up. You’ve tried various diets with limited success.
- You can either create the rest of your backstory or use your own experiences.

**Case 4: Patient Role**

- You are a 30-year-old marketing executive and have been suffering with chronic migraine for the last three years.
- You had migraine attacks on 15 or more days a month. Migraine attack are also associated with a host of classic symptoms, such as nausea and light or sound sensitivity (both known as photophobia), vision problems like flashing lights.
- You have already taken a few different over the counter pain relievers and prescription drugs but nothing seems to be working on its own without bringing back the illness. The pain along with other symptoms are getting in the way of your work and general happiness.
- You have come to the community health centre today because your migraine attacks are occurring more often and becoming worse in intensity over the past few weeks. You are worried about how this is affecting your life.
- Since the clinic was little busy, the nurse told you to wait for your turn. But first, you are going to be interviewed by a first-year medical student before the doctor consultation.
- You are scared of the long-term effects of chronic migraine might be doing to your brain, and if you will ever find a treatment plan that works. Use mild expressions of frustration or concern (like rub your temples, frown deeply and speak with a tone of frustration). If the medical student responses: “It sounds like your migraines are really affecting your life, and it’s understandable that you’re feeling frustrated. What have you tried so far, and let's see what else we can explore together to sort out the issues." --> answer, with a blend of frustration and qualified optimism.
- You worry about the chance that you might have to take a daily preventive migraine medication and any side effects or actions it may cause. And you are afraid, that all of your sick days cause the deadlines to be delayed. But unless the medical student directly inquires about your worries or how you are dealing with migraines everyday, you don’t mention these thoughts to them.
- You have migraine history running in the family where even your mother and elder sister complain of migraines. You used to be fine, but you find that sleep and stress make migraines strike. Maybe you've tried to cope with your triggers, but the migraines are still there.
- You can fill in the rest of your background or with experiences.
